# Supplementary material for: Dynamics of the State of Arterial Stiffness as a Possible Pathophysiological Factor of Unfavorable Long-Term Prognosis in Patients after Coronary Artery Bypass Grafting
Source: Biomedicines. 2024 May 6;12(5):1018. doi: 10.3390/biomedicines12051018 (PMC11117762; doi:10.3390/biomedicines12051018)
Supplement: Supplementary file 1 [file biomedicines-12-01018-s001.zip › biomedicines-2915393-supplementary.pdf]

Table S1.The structure of deaths during 10-year follow-up in groups with improved CAVI or with worsened CAVI

	**Group with improved CAVI (n=94)**	**Group with worsened CAVI (n=116)**	**P-value**
All cause death (n,%)	14 (15.8)	32 (27.6)	0.029
Death from cardiac causes (n,%)	9 (9.6)	17 (14.7)	0.266
Death from non-cardiac causes (n,%)	5 (5.3)	14 (12.0)	0.089
Death of malignancies (n,%)	2 (2.1)	9 (7.8)	0.07
Covid 2019 (n,%)	1 (1.,1)	3 (2.6)	0.422
The cause of death is unknown (n,%)	0 (0)	1 (0.9)	0.367

Table S2. Comparisons of survival between groups with improved CAVI or with worsened CAVI using Log Rank, Breslow, Tarone-Ware tests

Overall Comparisons			
	Chi-Square	df	Sig.
Log Rank (Mantel-Cox)	2.566	1	0.109
Breslow (Generalized Wilcoxon)	3.275	1	0.070
Tarone-Ware	3.047	1	0.081
Test of equality of survival distributions for the different levels of groups with improved CAVI and with worsened CAVI			

Table S3. Comparisons of event-free survival between groups with improved CAVI or with worsened CAVI using Log Rank, Breslow, Tarone-Ware tests

Overall Comparisons			
	Chi-Square	df	Sig.
Log Rank (Mantel-Cox)	4.511	1	0.034
Breslow (Generalized Wilcoxon)	5.571	1	0.018
Tarone-Ware	5.178	1	0.023
Test of equality of event-free survival distributions for the different levels of groups with improved CAVI and with worsened CAVI			
